# Offending behaviour and mental ill-health among young people: Reducing recidivism requires integration with youth mental health care

**DOI:** 10.7189/jogh.14.03001

**Published:** 2024-02-16

**Authors:** Simon M Rice, David G Baker, Rosemary Purcell, Andrew Chanen

**Affiliations:** 1 Orygen, Melbourne, Victoria, Australia; 2 Centre for Youth Mental Health, The University of Melbourne, Melbourne, Victoria, Australia

Adolescence marks a challenging confluence, with the frequent onset of both offending behaviour and mental ill-health. Although repeated offending behaviour is limited to a small minority of young people, it is disproportionately associated with poorer life course outcomes, social costs, and community concerns. Each year, around 30 million people are incarcerated in the world’s prisons [1]. Based on 2019 United Nations (UN) data from 121 countries, 261 000 adolescents aged ≤17 years are detained on any given day, and a further 410 000 are held in detention or pre-sentence remand annually [2]. Due to inaccurate and inconsistent reporting globally, these figures underestimate the scope of the problem. Most youth offending involves minor offences (e.g. property damage, theft); however, youth violence is the fourth leading course of death globally among ten to 29-year-olds, with 83% of victims being young males [3]. For each death due to youth violence, approximately 20 to 40 additional young people will require hospital-based treatment due to injuries sustained [4]. Addressing the factors contributing to the causes and desistance of youth offending is a global health imperative.

In this viewpoint, we present a rationale for integrating the advances in youth mental health [5] within the management of youth offending internationally. We contend that commensurate health and community benefits are achievable through integrated early detection and intervention models for mental ill-health among justice-involved young people.

## FORENSIC YOUTH MENTAL HEALTH – AN EMERGING FIELD

The age-crime curve suggests that a minority of youth with an offending history will demonstrate persistence into adulthood [6]. However, young people who engage in offending behaviour experience a disproportionately high prevalence of mental ill-health, increasing with deeper involvement in the justice system, with some estimates exceeding 95% of youth forensic cases experiencing mental ill-health [7]. A large proportion of youth offending behaviour co-occurs with problematic substance use or dependence (ranging from 25% to 67%) [8], which are rightfully seen as mental health issues. Neurodevelopmental impairments and disorders are also over-represented among young people with an offending history. Mental health help-seeking among justice-involved young people is significantly lower than that observed for same-aged young people among the general population [9]. Therefore, integrated models of care are needed to engage justice-involved young people in evidence-based mental health care. The emergent, but long-overdue, field of forensic youth mental health can bridge the gap between offending and mental ill-health, thereby addressing mental ill-health as a common and fundamental feature among justice-involved youth rather than an exceptional characteristic.

Where a young person engages in offending behaviour co-occurring with, or consequent upon, mental ill-health, the two are typically disentangled and managed separately. Offending behaviour is addressed through a judicial or legal process, typically including diversion, community-based supervision pathways, or custody. In contrast, mental ill-health is often unnoticed or disregarded. Where resources permit, external, contracted mental health services provide ‘inreach’ into community or custodial justice settings. However, these services are rarely integrated with the justice environment or sufficient for the needs of this population. In particular, the time-limited opportunity to engage with this hard-to-access and engaging population is missed, and care is usually fragmented across custodial or community settings, different providers, and geographical regions.

Forensic youth mental health also faces two structural challenges. First, the universal problem of global disparities in access to mental health services. Second, the structural legacy of ‘juvenile’ or ‘youth’ justice systems that have failed to adapt to the compelling developmental evidence that the capacity for criminal responsibility commences around age 14 [10], and that the transition from childhood to adulthood extends well into the third decade of life [11]. The former results in rerouting young people from social care into custodial settings. The latter results in the majority of incarcerated young people being held in ‘adult’ prisons, where they are unlikely to receive developmentally appropriate care or access to early intervention.

Ineffectively addressing the mental health needs of justice-involved youth may disrupt key processes in the development of adult role functioning. These include the development of prosocial relationships and support networks, educational and employment opportunities. It also increases the likelihood of entrenched offending, antisocial behaviour, marginalisation, and other social and financial costs. We argue that gains can be made even in settings with comparatively limited resources.

Justice-involved young people experience significant psychosocial vulnerabilities that are often intertwined with complex social care needs. Increasingly, programs are seeking to divert young people away from custodial sentences, and there is a growing emphasis on integrating diversion programs with mental health care [12]. A recent review of 31 diversion programs by Lindquist-Grantz and colleagues reported evidence for improvements in recidivism and psychosocial outcomes [13]. However, all reviewed programs from the Lindquist-Grantz review were from high-income countries, and only eight of these spanned the youth age range, albeit limited to age 21. Such diversion programs offer the opportunity for socio-culturally informed interventions to be delivered.

There is an inverse relationship between the level of offence and the number of offenders. For example, high rates of youth delinquency but fewer youth perpetrating violence. Delinquency rates are higher in societies experiencing political change [14]. The rates of youth homicide decreased in most countries between 2000–19 [15]. Internationally, for every 100 crimes recorded, 4.1 juveniles are alleged to have offended, 2.2 are prosecuted, and 1.4 are convicted [16].

## SOCIO-CULTURALLY INFORMED INTERVENTIONS

Adaptation of contemporary mental health care models that integrate culturally appropriate and informed approaches [17] are increasingly occurring across low- and middle-income countries [18]. We call for intensified and expanded efforts here. Recent exemplars include promising findings from studies testing cognitive behavioural therapy among youth offenders involved in drug-related crime in Indonesia [19], narrative exposure therapy for violent youth offenders in South Africa [20], and a cognition-focused intervention among Iranian youth with offending histories [21]. In our own country, Indigenous Australian youth in 2020–21 were 16 times more likely to be under criminal justice supervision than non-Indigenous Australian youth on an average day [22], and Indigenous Australians do not access community-based mental health services prior to imprisonment at a level commensurate with their need [17]. Cultural continuity is widely recognised as an essential component of positive identity development for Indigenous youth and an important aspect of psychosocial care provision [23].

Youth forensic mental health approaches must also integrate an understanding of masculinities [24], given the disproportionate representation of young men in justice-involved populations. Young men are at a markedly higher likelihood of justice system involvement (3.7 times more likely to be arrested; 3.9 times more likely to be sentenced) relative to female peers [25]. Cultural norms associated with the expression of masculinities, including perceived status (or lack thereof), feature strongly among male youth in custodial settings. Youth offending is often a group phenomenon, and the masculinities that young men embody contribute to group affiliation and the status this affords. Such norms powerfully and negatively impact the willingness of young men to engage in mental health care [26], delaying help-seeking, usually until symptoms or impairment are severe.

It is common in youth mental health that delays in treatment are associated with worse outcomes. Socio-culturally tailored interventions offer a vehicle for engaging this population in mental health care. To achieve this, however, mental health clinicians will likely need to modify their approaches to treatment [24] (i.e. being more accepting of client defensiveness or mistrust, being open to working with symptom complexity, and acceptance of a gradual the pace of change).

## ADVANCING YOUTH FORENSIC TREATMENT

Urgent innovation is needed to quantify and describe the full extent of the challenges facing us and to develop novel and effective interventions. Prospective studies incorporating long-term post-release follow-up of justice-involved youth will contribute to an improved understanding of reconviction [27] and subsequent refinement of leading explanatory forensic models such as Risk-Need-Responsivity [28]. In the UK, program and policy progress has been made based on the Child First, Offender Second [29] approach, and we emphasise that models prioritising the best interests of youth offenders are urgently needed (i.e. Youth First, Offender Second). The imperative for early detection and intervention is paramount (i.e. ideally at a first encounter with the justice system). Recent data from a regional Forensic Child and Adolescent Mental Health Service in the UK shows that lowering the threshold for specialist forensic involvement can widen access, offering greater coordinated community input, increased diversion and reduced need for secure youth justice provision [30]. It is essential that such programs ensure proportional engagement with higher risk and marginalised populations (e.g. culturally and linguistically diverse youth), which is dependent on successfully building and leveraging connections, local partnerships and trusting relationships, including with elders among the community [17].

## THE ROAD AHEAD

Internationally agreed minimum standards for treating prisoners enshrine protections for mental health [31] and principles for the administration of youth justice [32]. Sadly, many countries do not always guarantee these basic protections. Most urgent are those low-income jurisdictions that still imprison children and adolescents within adult custodial settings, and high-income jurisdictions that imprison children as young as ten years old. As a minimum, we call for international legal systems to develop greater awareness of youth mental-ill health as a precipitating and perpetuating factor for youth offending and consider ways by which effective youth mental health treatments could be offered across the spectrum of forensic involvement.

As early as 1971, the integration of youth psychiatry and the law (i.e. Toronto’s Family Court Clinic) offered youth presenting to court psychosocial support delivered by mental health clinicians, including assessment, mental health consultation and referral services [33]. Broadening such integrated mental health supports beyond court to consider ‘through-care’, spanning first encounters with police [12,34] to supported post-custody release [35] remains a promising approach to explore and evaluate.

While custodial models of specialist care integrating multidisciplinary youth mental health expertise are now emerging – e.g. the Orygen Forensic Youth Mental Health program in Melbourne, Australia [24] – such programs are resource-intensive, skewed toward custodial (rather than community-based) settings, and are not yet scalable beyond high-income countries. To support the universal need for specialist forensic youth mental health programs, lower-income countries can embark on education/training programs for police and prison staff regarding youth mental health (e.g. symptom/disorder presentation and behavioural impacts in custody), development of good practice guidelines for liaison with health providers [36], legislative changes to the age of criminal responsibility, and ending the incarceration of young people in adult prisons.

Mental health care should be an enshrined right for justice-involved youth, and youth justice involvement represents a golden opportunity for engagement with youth mental health services [12]. Justice-involved youth should have equity of access to evidence-based mental health intervention [1,31], which is an essential aspect of recidivism reduction and supporting the transition to adulthood and a life of contribution and meaning. Specialist forensic youth mental health services offer a major advantage in providing care coordination and continuity for young people as they move through the justice system [24]. This spans routine mental health screening at first contact through to post-release linkage and inter-agency planning. Meaningfully shifting the trajectory of those youth likely to progress into the adult prison system and long-term disability, every contact with the justice system should be exploited, and psychosocial challenges must become a key focus of recovery.
